# Effect of low salicylate diet on clinical and inflammatory markers in patients with aspirin exacerbated respiratory disease – a randomized crossover trial

**DOI:** 10.1186/s40463-021-00502-4

**Published:** 2021-04-23

**Authors:** Leigh J. Sowerby, Krupal B. Patel, Crystal Schmerk, Brian W. Rotenberg, Taciano Rocha, Doron D. Sommer

**Affiliations:** 1 Department of Otolaryngology – Head & Neck Surgery, Schulich Medicine & Dentistry, Western University, London Health Sciences Centre, St. Joseph’s Hospital, London, Ontario Canada; 2 Department of Medicine, Western University, London, ON Canada; 3 Division of Otolaryngology – Department of Surgery, McMaster University, Hamilton, ON Canada

**Keywords:** Chronic rhinosinusitis, Asthma, Aspirin sensitivity, Immune system diseases, Nasal polyps

## Abstract

**Background:**

Aspirin-exacerbated respiratory disease (AERD) is characterized by eosinophilic rhinosinusitis, nasal polyposis, and bronchial asthma, along with the onset of respiratory reactions after the ingestion of nonsteroidal anti-inflammatory drugs (NSAIDs) or acetylsalicylic acid (ASA). In addition to the therapeutic routines and surgical options available, a low dietary intake of food salicylate has been suggested as adjunctive therapy for this condition.

This study aimed to assess the influence of a short-term low salicylate diet on inflammatory markers in patients with AERD and whether that would result in symptomatic improvement.

**Methods:**

Prospective study with randomization to either a high or low salicylate diet for 1 week, followed by cross-over to the other study arm. Participants were asked to record their dietary salicylate for each week of the study. Urinary creatinine, salicylate and leukotriene levels were measured at the time of recruitment, end of week one and end of week two and the SNOT-22 questionnaire was filled out at the same time points.

**Results:**

A total of seven participants completed the study. There was no statistical difference in the urinary salicylate and leukotriene levels between the two diets; nevertheless, participants on low salicylate diet reported improved SNOT-22 symptoms scores (*p* = 0.04), mainly in the rhinologic, ear/facial, and sleep dysfunction symptom domains. In addition, these last two domains outcomes were more significant than the minimal clinically important difference.

**Conclusions:**

A short-term low salicylate diet may not result in biochemical outcomes changes but seems to provide significant symptomatic relief for patients with AERD.

**Trial registration:**

NCT01778465 (www.clinicaltrials.gov)

**Graphical abstract:**

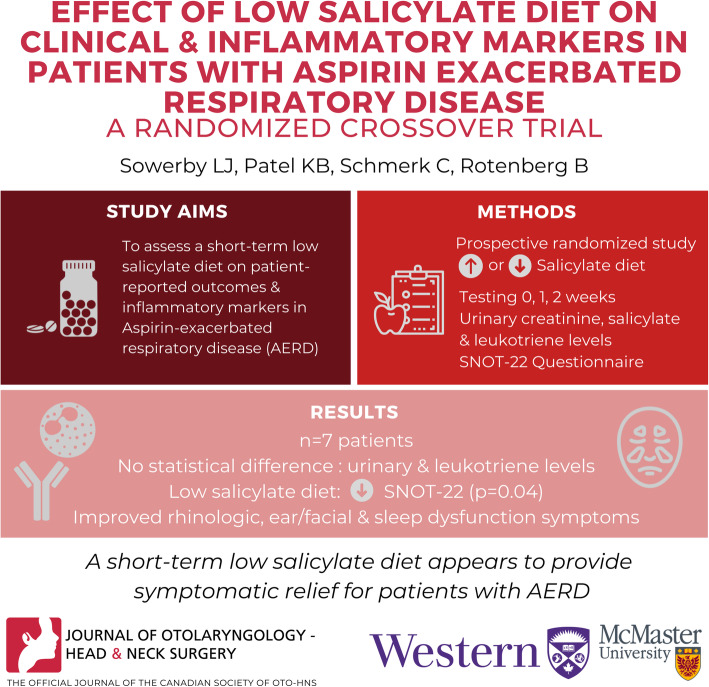

**Supplementary Information:**

The online version contains supplementary material available at 10.1186/s40463-021-00502-4.

## Background

Aspirin exacerbated respiratory disease (AERD) is an acquired condition with a median age of onset around 30 years, that consists of bronchial asthma, chronic rhinosinusitis with nasal polyposis and hypersensitivity to acetylsalicylic acid (ASA) [1, 2]. In AERD patients, acetylsalicylic acid (ASA) intolerance is a non-allergic hypersensitivity reaction without immunoglobulin E (IgE) involvement. These patients have recalcitrant chronic rhinosinusitis with nasal polyposis (CRSwNP), often requiring frequent surgical intervention, systemic corticosteroid therapy and aspirin desensitization [2, 3].

The pathophysiology of AERD has not been entirely elucidated; however, it is thought that increased expression of 5-lipoxygenase and leukotriene C4 synthase genes which results in downstream inflammatory activation, including mast cells and eosinophils. Altered metabolism of arachidonic acid results in an imbalance of prostaglandins (PG) and leukotrienes (LT), eliciting increased levels of cysteinyl leukotrienes (CysLT) and decreased levels of prostaglandin E2 (PGE2) [4].

Patient avoidance of non-selective COX inhibitors, medical and surgical procedures to address the underlying bronchial asthma and CRSwNP and ASA desensitization therapy, are standard for AERD management [5].

Dietary modifications have been used as an alternative to modulating the inflammatory status in patients with chronic diseases, such as CRS [6, 7]. It is estimated that daily intake of dietary salicylate can, occasionally, exceed the equivalent blood salicylate level of patients who take low-dose aspirin (i.e. 81 mg/day); however, salicylate bioavailability in food still warrants further investigation [8, 9]. Nonetheless, the potential influence of dietary components for AERD patients should not be underestimated. Many of these patients are prone to present upper and lower-airway hypersensitivity reactions to alcoholic beverages, in particular beer and red wine [2]. Also, extracts of plants such as poplar, myrtle, willow and meadowsweet, which are rich in salicylates, have been used for millennia due to analgesic and antipyretic effects. Willow and meadowsweet were the first sources of isolated salicylic acid, early in the nineteenth century [10].

Our group has previously demonstrated that adopting a low-salicylate diet can provide a significant reduction in sinonasal symptoms, along with improved nasal endoscopy scores, in comparison to regular dietary intake [11, 12]. Despite the encouraging results of modifying dietary salicylate intake, the biochemical effect of this intervention has not yet been evaluated with subjective sinonasal symptom correlation.

Therefore, the objective of this study was to evaluate the inflammatory status of AERD patients under both low and high salicylate diets, testing the hypothesis that a low salicylate diet would decrease urinary salicylate levels and inflammatory markers, as well as improve the participants’ subjective sinonasal symptoms.

## Materials and methods

This study was a prospective randomized, cross-over study assessing biochemical changes in the levels of urinary salicylates and urinary cysteinyl leukotrienes (CysLT), in relation to dietary salicylate intake in subjects with AERD. This was approved and reviewed by the research ethics board at Western University (London, ON, Canada – REB #103330) and was registered with clinicaltrials.gov (registration #01778465). The Sino-nasal Outcome Test (SNOT-22), categorized into five symptom domains, was used to assess, as a secondary outcome, the participants’ symptoms [13].

Adults, 18 years and older, were assessed in the Department of Otolaryngology -Head and Neck Surgery at Western University. Patients with a history of surgery for chronic rhinosinusitis with nasal polyposis, confirmed asthma, and a documented history of a significant respiratory sensitivity reaction to ASA or ibuprofen were invited to participate in the study. Informed consent was obtained from all individual participants before inclusion. Exclusion criteria included a history of cystic fibrosis, immunodeficiency, recent endoscopic sinus surgery (within the past 6 months), and treatment with a course of oral corticosteroids within 3 months previous to enrollment. None of the enrolled patients were taking leukotriene receptor antagonists.

On the first clinic visit, participants were randomized to high (HS) or low (LS) salicylate diets, using an online randomization tool. They were briefed on a salicylate diet regarding the salicylate contents of foods, and a handbook was given to them comprising a detailed list of the salicylate contents of different foods (Supplement 1). The low-salicylate diet consisted of avoiding foods with high (> 0.5 mg/portion) salicylate content and trying to consume foods with low (0.01 to 0.09 mg/portion) salicylate content for 1 week. Conversely, the high salicylate diet consisted of consuming mainly foods from the high salicylate group during the high salicylate diet week [9, 14]. After 1 week of diet (1st assigned one), patients crossed over to the opposite diet for the following week.

Outcome variables were assessed at the enrollment visit, 1 day after the completion of the first week (1st diet), and 1 day after the second week (2nd diet). At each of these time points, participants filled out the SNOT-22 questionnaire and had urinary samples collected. Additionally, participants kept a diet diary, in which they included a record of their everyday salicylate dietary component intake to help them keep track of their salicylate diet intake and comply with their assigned diets.

### Outcomes

#### Urinary biomarkers

The urine samples were aliquoted into five different 1 ml aliquots and stored at − 80 °C until processed. Urinary creatinine (Cr) was determined using the standard core laboratory protocol (enzymatic method). After thawing, samples were centrifuged at 3000 rpm for 10 min at 4 °C. Cysteinyl leukotrienes were measured in the supernatant of each sample using an ELISA kit (Cayman Chemical Co., Ann Arbor, MI) per manufacturer’s protocol. Briefly, 50 μL of supernatant, CysLT acetylcholinesterase conjugated tracer, and CysLT monoclonal antibody were added to each well of a 96-well plate. Following overnight incubation at 4 °C, the wells were emptied and washed five times with wash buffer. To develop the assay, 200 μL of Ellman’s Reagent was added to each well, and the plate was placed on an orbital shaker in the dark for 90 min. The absorbance was measured at a wavelength of 412 nm. Sample concentrations were calculated according to the manufacturer guidelines.

Urinary Salicylic and Salicyluric acids were measured based on the methods of Baxter et al. [15].

#### Sino-nasal outcome test (SNOT-22)

Study participants responded to a subjective symptom outcome test (sino-nasal outcome test; SNOT 22), a 22-item validated questionnaire that quantifies the severity of sinonasal symptoms (Washington University, St. Louis, MO). Each one of the 22 items in the test are graded from zero to five; the higher the score, the more severe the disease; additionally, it is subdivided into five distinct symptom domain scores: the rhinologic symptoms domain (range: 0–30), extra-nasal rhinologic symptoms domain (range: 0–15), ear/facial symptoms domain (range: 0–25), psychological dysfunction domain (range: 0–35), and the sleep dysfunction domain (range: 0–25) [13].

#### Data analysis

The analysis was conducted using SPSS for Windows (version 20.0, Chicago, IL). In this study, the variables are presented as median and interquartile ranges, and those values were used for the hypothesis analysis. Three time points were predetermined for outcomes analysis (e.g. baseline measurements, post HS, and post LS). Furthermore, the effect of the interventions on each variable (Δ) was calculated by subtracting the individual baseline values of each participant from their Post value (i.e. post - baseline). Individual results were then compiled, and a group median was created.

Post diet outcome variables values were compared to their baseline values using the Wilcoxon signed-rank test in order to mimic the comparison between the group intervention to a regular diet. Moreover, the comparisons between the effect of each intervention were made by using the Mann-Whitney test. Both test results are expressed in terms of significance (2-tailed null hypothesis and significance levels *p* < 0.05) and effect size r, utilizing the equation “*r* = Z/ √*N*” [16].

## Results

**Fig. 1:**
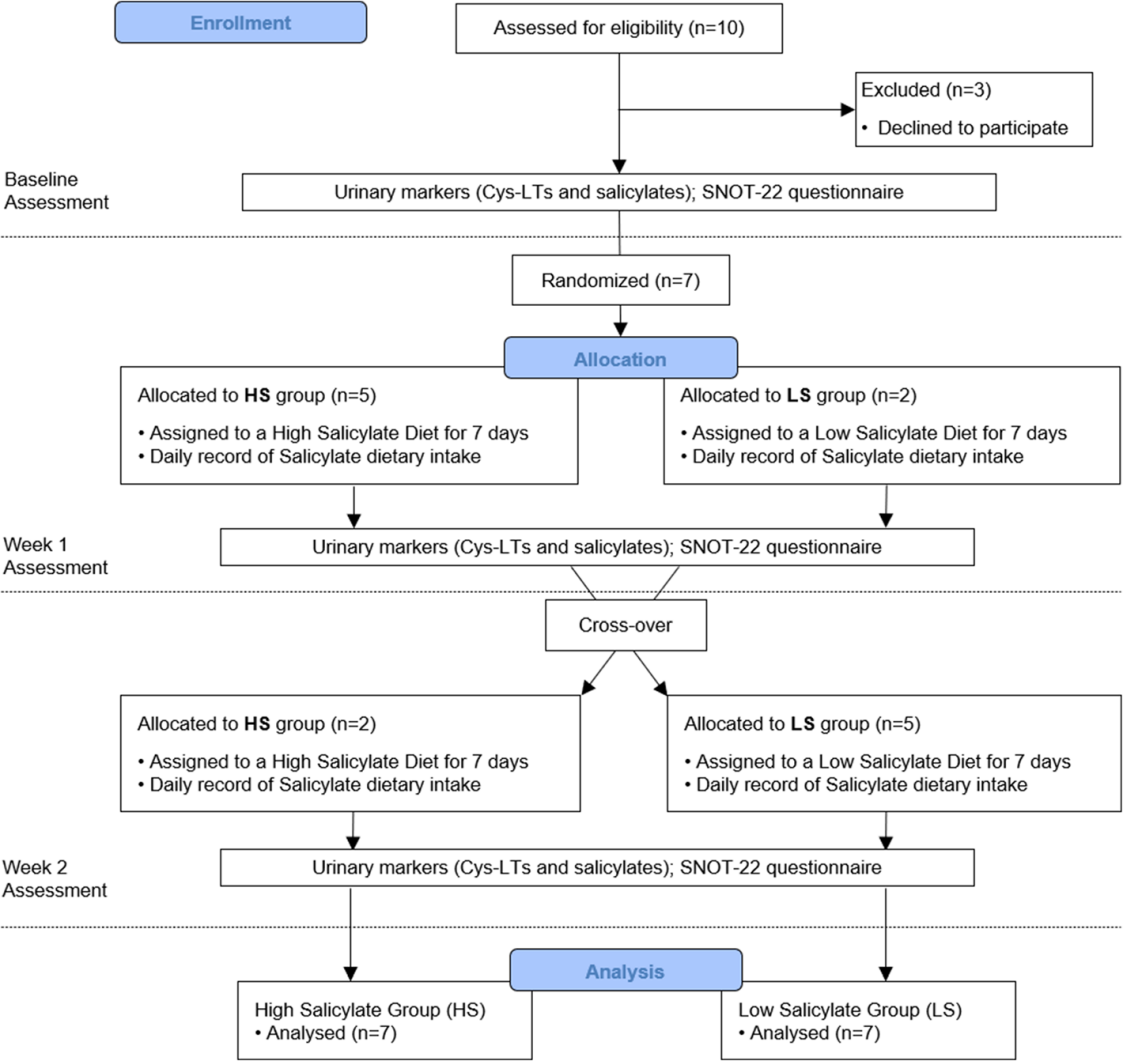
Study Flow Diagram

A total of ten potential participants were screened for this study. After consideration, three of them declined participation before the first week of diet; therefore, seven participants completed this study (5 men and 2 women), average age 55 (SD13.1). The study flow chart details the sequence of the interventions and evaluations throughout the study (Fig. 1).

### Urinary biomarkers

Except for creatinine levels, urinary biomarkers of both groups, did not yield statistically significant differences over the study (Table 1). In the intragroup analysis (i.e. post x baseline), HS presented a significant increase in the urinary creatinine, whereas the LS remained unchanged.

**Table 1:** Participants biomarkers and symptom levels on both diets, high salicylate and low salicylate

	Baseline		HS				LS				
			Post		Δ High		Post		Δ Low		Δ High x Δ Low
	Median	IQR	Median	IQR	Median	IQR	Median	IQR	Median	IQR	*p*
Urinary SA	2.62	4.5	0.92	0.8	−1.7	3.0	0.6	3.4	−1.13	2.1	0.338
Urinary SU	37.9	45.6	23.82	54.3	−16.76	31.6	44.21	34.6	−7.35	19.3	0.482
Urinary CysLT	819.88	2649.6	771.99	1327.9	− 173.17	1065.2	874.05	1221.4	− 184.58	1518.9	0.949
Urinary Cr	10.3	11.3	*15.3	12.6	2.6	8.4	9.9	11.3	−0.3	11.1	0.655
SNOT - 22	66	40	64	40	7	15	*44	31	−10	24	**0.013**

*HS* High-Salicylate Diet, *LS* Low-Salicylate Diet, *SA* Salicylic Acid (ng/ml), *SU* Salicyluric Acid (ng/ml), *CysLT* Cysteinyl leukotrienes (pg/ml), *Cr* Creatine (mmol/L), *SNOT − 22* Sino-nasal outcome test, *Δ High* Post - Baseline of High-Salicylate diet group, *Δ Low* Post - Baseline of Low-salicylate diet group. * significance *p* ≤ 0.05 (Wilcoxon Test; baseline x post); Δ High x Δ Low = intergroup dietary effect comparison (Mann Whitney U test)

**Fig. 2:**
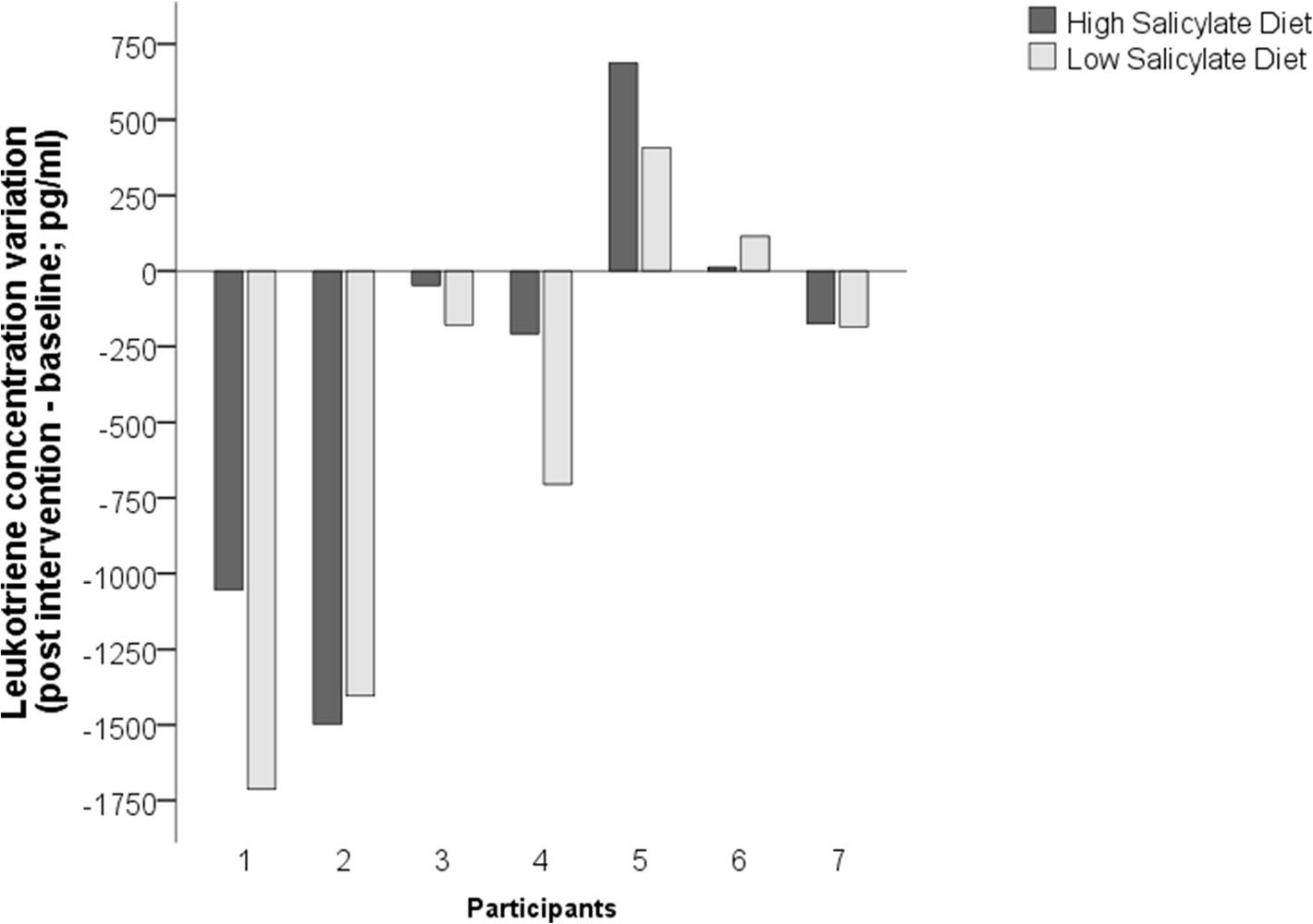
CysLT variation post High and Low Salicylate diets

The data variance within group participants was considerably high on both groups, especially on the CysLT deltas. The expression of that outcome, case by case, shows that most of participants (5/7) presented a reduction in the CysLT concentration, regardless of their dietary intake (Fig. 2). Despite the absence of statistical significance, the overall CysLT reduction was higher in the LS participants, compared to the HS.

### Sino-nasal outcome test (SNOT-22)

**Fig. 3:**
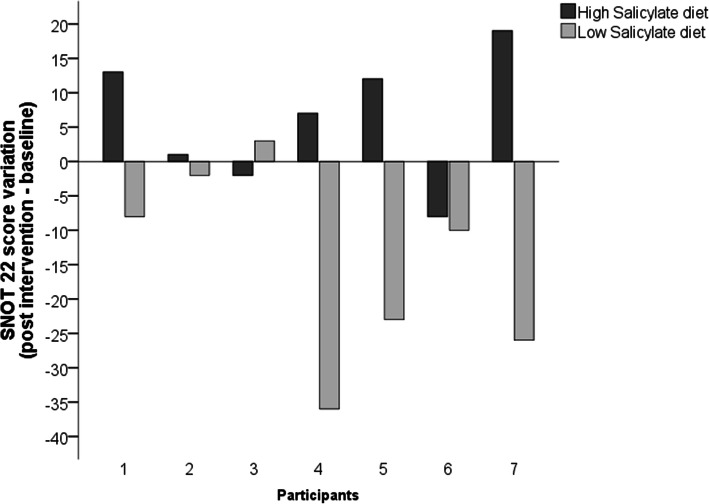
SNOT 22 score variation post High and Low Salicylate diets

The participants’ nasal symptoms after 1 week on LS demonstrated a statistically significant reduction of 22 points (*p* = 0.043; effect size *r* = − 0.53), compared to the baseline, while the group HS did not present significant improvement, on the intragroup analysis (Table 1). Furthermore, an intergroup analysis of both groups’ deltas (e.g. post - baseline) medians values showed that LS reduced significantly 10 points on the nasal symptoms test, whereas HS presented an increase in this variable (*p* = 0.013; effect size *r* = − 0.66). Expressed case by case, the difference between interventions on the SNOT 22 outcome is clearly identified where the LS was more effective on reducing the severity of the sinonasal symptoms, except for one participant, in comparison to the HS (Fig. 3).

**Fig. 4:**
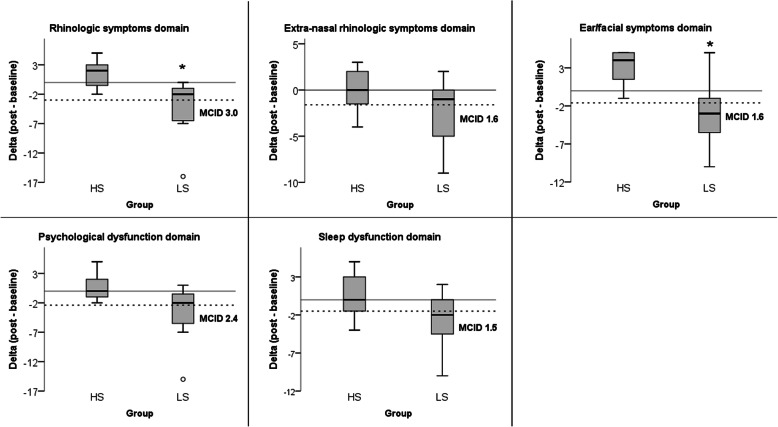
Inter-group analysis of SNOT 22 results in 5 domains. Dotted lines represent the minimal clinically important difference (MICD) for each domain [13]. Asterisks express the Mann-Whitney test with a 2-tailed significance ≤ .05

A segmented analysis of the nasal symptoms outcome into domains is presented in Fig. 4. All five domains contributed to the overall result reported on the paragraph above; however, only the Rhinologic (HS, median 2, IQR 7; LS, median − 2, IQR 16; *p* = 0.017, effect size *r* = − 0.63) and the ear/facial (HS, median 4, IQR 6; LS, median − 3, IQR 15; *p* = 0.02, effect size *r* = − 0.62) symptoms domains were statistically different between both groups.

The analysis of each domains’ results regarding the minimal clinically important difference (MCID), according to the literature [13], showed that on top of the effect presented by the LS group, two domains overcame their respective MCID (Fig. 4). The LS group median on the ear/face symptoms domain was − 3 (IQR 15), whereas the MCID for this domain is 1.6, and the sleep disfunction was − 2 (IQR 12), while its MCID is 1.5.

## Discussion

Our results showed that consuming a low salicylate diet for at least 1week resulted in a symptomatic improvement of subjective sinonasal symptoms in patients with AERD. However, the urinary leukotrienes did not correlate with these findings. Salicylates are ubiquitously present in modern diet and strict adherence to diet long-term may be challenging and pose difficulties in achieving a balanced diet. Some patients have commented that they find a less stringent version of the diet more achievable e.g. avoiding foods with high/very high salicylate content. Another successful strategy has been to follow the strict diet as an elimination diet, and add in desired foods after a month to evaluate impact on symptoms. Despite these limitations, a low-salicylate diet could be an adjunctive treatment for patients who have increased frequency and intensity of symptoms and are highly motivated to pursue alternative non-pharmacologic therapy.

AERD is among the more challenging presentations of CRSwNP to treat. Most patients require multiple surgeries and multifaceted medical management including topical saline irrigations, topical and systemic corticosteroids and aspirin desensitization [17, 18]. However, apart from requiring expertise in initiating the protocol, up to 30% of the patients on aspirin desensitization protocols report gastrointestinal side effects and discontinue therapy [19]. AERD patients often require multiple revision surgeries with some patients undergoing ten times as many surgeries as non-AERD patients with CRS [20, 21]. Additionally, the interval between surgeries is also decreased for AERD patients compared to non-AERD CRS patients. Monoclonal antibody therapy targeting type II inflammation is a promising development for use in CRSwNP and will change our treatment paradigm. These medications are, however, quite expensive and may not be an option for all patients.

Wood et al. [8] published a systematic review, which showed the average daily dietary salicylate intake for men is 4.42 mg/day and for women is 3.16 mg/day, [9] with even higher values in vegetarians. Interestingly, in the current study, urinary cys-LTs levels in both low-salicylate and high salicylate diet groups were reduced compared to baseline. Thus, it is possible that the participants already had a significantly high baseline salicylate intake; therefore, the effort to create an enhanced salicylate intake (i.e. high salicylate group), might have been compromised by participants’ pre-diet salicylate consumption levels. Additionally, participants might have been unable to alter their diet effectively, despite being advised to increase their dietary salicylate content when on high salicylate diet. Notably, six of the 7 patients continued on a modified low-salicylate diet after the study, as they found significant benefit from it, and were still continuing to follow the diet to some degree 6 months after participation in the research study.

Previous studies have shown that restricting dietary salicylates as an adjunct to treat AERD patients has demonstrated significant improvement in their symptoms [11, 12]. Our group’s previous multicenter study [11] with 30 participants undergoing a low salicylate diet for 6 weeks presented a median reduction of 15 points in the SNOT 22 score. In the present study, with only 1 week under a low salicylate diet, the participants demonstrated an overall reduction in the SNOT-22 scores of 10 points. Both a placebo and Hawthorne effect are realistic possibilities for this effect, but continued observance of the diet (with some allowances) is evidence to the contrary.

The symptomatic improvement presented in our study overcomes the minimal clinically important difference (MCID) of 9 points, described by Chowdhury et al. [13] in patients with chronic rhinosinusitis undergoing continued appropriate medical therapy. On the other hand, Phillips et al. [22] describe a MCID for medically managed CRS patients of 12, which is higher than the overall median value on the LS group. In this regard, none of these studies specify patients with AERD, making comparison to our study results a challenge. The symptomatic domain analysis of SNOT 22 is performed to segment the quality of life analysis into distinct sections, which can work as references for treatment guidance or therapy effectiveness analysis [13]. For patients with CRS, the psychological and sleep dysfunction domains demonstrate significant associations with productivity loss [23]. As such, there may be potential for the low salicylate diet to help AERD patients improve productivity and their overall quality of life.

The short duration of the diet was chosen based on the half-life of CysLTs and the difficulty with adherence to the restrictiveness of the low salicylate diet. It was hoped that by choosing 1 week, patients would be able to provide maximal adherence to both the HS and LS diets. Additionally, the half-life for salicylates is somewhere between 2 and 30 h. Therefore, a washout period was not included as measurements of both Cys-LTS and SNOT-22 was performed at the end of the week period. It is conceivable that the time period of investigating the diet may have been affected by the half-life of salicylates. The two previous studies investigating salicylate diet in AERD patients had a 6 week time course on the diet, compared to 1 week in the current study. Given the presented subjective symptom outcomes, 1 week under a low salicylate diet already provides AERD patients with overall symptomatic improvement.

Measurement of urinary LTS is possible using a variety of techniques. As mentioned, our study utilized an ELISA based methodology as per Baxter et al. [15]. Other reports suggest that utilizing a liquid chromatography followed by tandem mass spectrometry and normalizing to urinary creatinine levels may be a clinically valid technique in the assessment of urinary leukotrienes as a biomarker of inflammation in diseases such as AERD. It is possible that our results would differ using the aforementioned methodology [15, 24].

Lastly, the low number of patients is also a limitation of this study, but on top of its design as a proof-of-concept study, recruitment was challenging given the need for three weekly visits after enrollment, and agreement to strictly adhere to the salicylate diet. Future studies should continue to investigate the mechanistic processes of dietary salicylates and their role in AERD. Increased control and recording of food salicylate intake amount, along with periodic measurements of inflammatory biomarkers throughout the dietary period, and a follow-up time after the diet discontinuation, should be included as part of further studies.

## Conclusion

This study confirms the previous findings that a low-salicylate diet decreases AERD patients’ symptoms as measured by SNOT-22. There was reduction in the urinary cys-LTs levels in both groups, with some patients showing a reduction in Cys-LTs while on a LS diet. This was a proof of concept study again demonstrating subjective symptom improvement with reduction of nutritional salicylate intake in the treatment of AERD patients.

## Supplementary Information

**Additional file 1.** Salicylate-Free Diet Food Guide.

## Data Availability

Not applicable.
