# Supplementary material for: Effect of low salicylate diet on clinical and inflammatory markers in patients with aspirin exacerbated respiratory disease – a randomized crossover trial
Source: J Otolaryngol Head Neck Surg. 2021 Apr 23;50:27. doi: 10.1186/s40463-021-00502-4 (PMC8063291; doi:10.1186/s40463-021-00502-4)
Supplement: Supplementary file 1 — Additional file 1. Salicylate-Free Diet Food Guide. [file 40463_2021_502_MOESM1_ESM.pdf]

Salicylate-Free Diet Food Guide

The following is a listing of foods based on their salicylate content highlighting those which are compatible with a low-salicylate diet and those which should be entirely avoided. Please use this list to guide your diet choices and use it as a referral for the attached cookbook, as some of the recipes include ingredients which contain natural salicylates (SalicylateSensitivity, 2012)

Nuts and Seeds

	Low	Moderate	High	Very High
Poppy seed	Cashews	Desiccated coconut	Brazil nuts	Almond
	Hazelnuts	Peanut Butter	Macadamia nuts	Peanuts with skins on
	Pecan	Pumpkin seeds	Pine nuts	Water chestnut
	Sunflower seeds	Sesame seeds	Pistachio	
		Walnuts		

Vegetables

Negligible	Low	Moderate	High	Very High
Bamboo Shoots	Bean Sprouts	Asparagus – tinned	Alfalfa sprouts	Canned Green Olives
Beans (dried – not borlotti)	Borlotti beans	Aubergine – peeled	Artichoke	Capsicum
Cabbage (green or white)	Brussels sprouts	Beetroot	Aubergine with peel	Champignon
Celery	Cabbage – red	Black Olives	Broad bean	Chicory
Green Split peas	Cauliflower	Canned Asparagus	Broccoli	Chili peppers
Lentils (brown)	Chickpeas	Carrot	Canned black olive	Courgette
Lentils (red)	Chives	Fresh Tomato	Cucumber	Endive
Lettuce (iceberg)	Choko	Frozen Spinach	Cucumber	Gherkin
Peas (dried)	Fresh Asparagus	Lettuce (other than iceberg)	Eggplant	Hot pepper
Potato (old/white and peeled)	Green Beans	Marrow	Fresh Spinach	Peppers
Swede	Green Peas	Mushrooms	Okra	Radish
	Leek	Parsnips	Radish	Tomato

	Mung bean sprouts	Potato (new and red Pontiac)	Sweet Potato	Tomato Products
	Onion	Pumpkin	Water chestnut	Water Chestnut
	Potato (white with peel)	Snow Peas	Watercress	
	Shallots	Sprout	Zucchini	
	Yellow split peas	Sweet corn		
		Turnip		

Fruits

Negligible	Low	Moderate	High	Very High
Banana	Apple – golden delicious	Apple – red delicious	Apple – all other varieties	All dried Fruits
Canned Pear****	Nashi Pears	Canned or dried Fig	Canned Morello cherries	Apricot
Lime	Papaya	Canned pear****	Cantaloupe	Avocado
Pear (ripe and peeled)	Paw Paw	Custard apple	Grapefruit	Blackberry
	Tamarillo	Lemon	Kiwi fruit	Blackcurrant
		Loquat	Lychee	Blueberry
		Mango	Mandarin	Boysenberry
		Passion fruit	Melons	Cherries – all other kinds
		Pear (w/ peel)	Mulberry	Cranberry
		Persimmon	Nectarine	Currant
		Pomegranate	Peach	Date
		Rhubarb	Sugar banana	Grape
			Watermelon	Guava
				Loganberry
				Orange
				Pineapple
				Plum
				Prune
				Raisin
				Raspberry
				Redcurrant
				Rock melon
				Strawberry
				Sultana
				Tangelo
				Tangerine
				Youngberry

***Canned Pear has negligible amounts of salicylate if it is in sugar syrup, but if it is in natural juices/syrups, it has a moderate amount of salicylate as these often contain some peel, which contains salicylates

Sweets

Negligible	Low	Moderate	High	Very High
Carob	Caramel****	Molasses		Chewing gum
Cocoa	Golden Syrup	Raw Sugar		Fruit flavors
Homemade caramel****	Malt Extract			Honey
Maple Syrup				Honey flavors
White Sugar				Jam (except pear, preferably homemade)
				Liquorices
				Mint flavored sweets
				Peppermints

*** Caramel can be made from just sugar and water or sugar and milk – both are salicylate free and therefore caramel is generally alright. However, store bought caramel can have a variety of additives and added flavorings that do contain salicylate – so be careful when buying/eating caramel and be sure to read the ingredients

Seasonings, Condiments, Toppings& Sauces

Negligible	Low	Moderate	High	Very High
Golden Syrup	Apple Butter (only if homemade from acceptable varieties of apples)	Fresh Coriander Leaves (also known as Chinese parsley)	All Spice	Aniseed
Malt Vinegar	Chives	Horseradish	Bay leaf	Basil
Maple Syrup (pure)	Fennel – dried	Mayonnaise	Caraway	Black pepper
Salt	Garlic		Cardamom	Cayenne
	Parsley (except Chinese parsley also known as coriander)		Cinnamon	Celery powder
	Saffron		Cloves	Chili flakes
	Shallots		Coriander	Chili Powder
	Soy Sauce (if free of spices)		Ginger	Cider Vinegar
			Mixed herbs	Commercial Gravies & Sauces
			Mustard	Cumin
			Pimiento	Curry
				Dill
				Fenugreek

				Fish, meat, and tomato pastes
				Garam masala
				Ginger
				Honey
				Jam/Jelly (all commercial varieties – you can make your own from acceptable ingredients)
				Liquorices
				Mace
				Marmite
				Mint
				Mustard
				Nutmeg
				Oregano
				Paprika
				Peppermint
				Rosemary
				Sage
				Tabasco
				Tarragon
				Thyme
				Turmeric
				Vegemite and other Yeast Extracts****
				White pepper
				White Vinegar
				Wine Vinegar
				Worcester Sauce

**** Yeast extracts are high but they are different from the yeast used in baking. Baking Yeast is generally ok

Fats & Oils

Negligible	Low	Moderate	High	Very High
Butter	Ghee	Almond Oil	Copha	Coconut Oil
Canola Oil		Corn Oil	Sesame oil	Olive Oil
Margarine****		Peanut Oil	Walnut Oil	
Safflower Oil				
Soy Oil				
Sunflower Oil				

**** Only if it's made from vegetable oil or canola oil

Grains

Negligible	Low	Moderate	High	Very High
Barley			Breakfast cereals that include fruit, nuts, honey or coconut	
Buckwheat			Corn/maize cereals	
Millet			Cornmeal	
Oats (plan)			Flavored breakfast cereals	
Rice			Maize	
Rice cereals (plan)			Polenta	
Rye				
Wheat				

Meats

Negligible	Low	Moderate	High	Very High
Beef	Liver		Fish canned in an unacceptable oil and/or with seasonings added	Processed luncheon meats (many are seasoned and thus contain salicylates)
Chicken	Prawns		Gravy made from prepared mixes (i.e. stock cubes/bouillon/meat extracts/etc.)	Seasoned meats (e.g. salami, sausages, frankfurters, and hotdogs)
Eggs	Shellfish			
Fish				
Lamb				
Organ meats				
Rabbit				
Sausage casing				
Scallops				
Tripe				
Veal				

***Most meat, fish and poultry are salicylate free, processed/seasoned often contain salicylates

Dairy

Negligible	Low	Moderate	High	Very High
Butter		Blue vein Cheese		
Cream				
Cheese (not blue vein)				
Milk				
Yoghurt (natural only)				
Ice Cream ****				
Rice Milk				
Goat Milk				
Soy Milk ****				
Tofu				

**** Be sure the read the label carefully on these things, they are ok only if they do not have any additives or added flavorings. With Ice cream it can be very difficult to find brands that make pure and plan ice cream without all the additives (though there are a few out there), I recommend just making your own.

Baking Supplies

Negligible	Low	Moderate	High	Very High
Arrowroot		Sesame seeds	Corn Syrup	
Corn starch (also known as corn flour in Australia)				
Golden Syrup				
Malt				
Malt extract				
Poppy seeds				
Rice Flour				
Rye Flour				
Sago				
Soy Flour				
Sugar				
Sugar (brown, castor, granulated, icing, powdered)				
Tapioca				
Wheat Flour				

Commercial Snacks

Negligible	Low	Moderate	High	Very High
Plain potato chips (read the	Apple chips (only if	Popcorn	Chewing gum (all flavors)	

ingredients list)	homemade from acceptable varieties of apples)	Popping corn	Fruit flavored candy, gelato, ices, popsicles, sherbet, sorbet, and sweets	
			Licorice/liquorices (all flavors)	
			Mint/peppermint/wintergreen flavored candy/sweets	
			Pickles (and anything pickled)	

Beverages

Negligible	Low	Moderate	High	Very High
Decaffeinated Coffee	Dandelion coffee	Coco cola	regular coffee	
milk (goat and cow)	Store bought pear juice	Rose hip tea	all teas	
Ovaltine		Rose hip syrup	Cordials and fruit flavored drinks	
homemade pear juice			fruit and vegetable juices	
soy milk				
rice milk				
water				

Alcohol

Negligible	Low	Moderate	High	Very High
Gin		Cider	Liquor	
Whiskey		Beer	Port	
Vodka		Sherry	Wine	
		Brandy	Rum	

Ingredients to Avoid Within Products

Salicylate, Salicylic Acid, chemicals with the syllables SAL, CAMPH or MENTH, balsam & bisabol, octisalate, homosalate, mexoryl, meradimate, bioflavinoids, quercetin, hesperiden or rutin (FMT, 2012)

References

SalicylateSensitivity (2012). Salicylate Sensitivity Food Guide. www.salicylatesensitivity.com [Obtained Aug 26 2012].
